# Development of a Time Projection Chamber Readout with Hybrid Pixel Sensors for Beam Monitoring

**DOI:** 10.3390/s24082387

**Published:** 2024-04-09

**Authors:** Yingdong Song, Haibo Yang, Yuezhao Zhang, Jianwei Liao, Yanhao Jia, Peng Ma, Yufeng Hou, Xiangming Sun, Hulin Wang, Haisheng Song, Chengxin Zhao

**Affiliations:** 1 College of Physics and Electronic Engineering, Northwest Normal University, Lanzhou 730070, China; 17340798253@163.com; 2 Institute of Modern Physics, Chinese Academy of Sciences, Lanzhou 730000, China; yanghaibo@impcas.ac.cn (H.Y.);; 3 Advanced Energy Science and Technology Guangdong Laboratory, Huizhou 516003, China; 4 School of Nuclear Science and Technology, University of Chinese Academy of Sciences, Beijing 100049, China; 5 College of Physical Science and Technology, Central China Normal University, Wuhan 430000, China

**Keywords:** beam monitor, data acquisition, pixel detector, readout electronics

## Abstract

To monitor the position and profile of therapeutic carbon beams in real-time, in this paper, we proposed a system called HiBeam-T. The HiBeam-T is a time projection chamber (TPC) with forty Topmetal-II- CMOS pixel sensors as its readout. Each Topmetal-II- has 72 × 72 pixels with the size of 83 μm × 83 μm. The detector consists of the charge drift region and the charge collection area. The readout electronics comprise three Readout Control Modules and one Clock Synchronization Module. This Hibeam-T has a sensitive area of 20 × 20 cm and can acquire the center of the incident beams. The test with a continuous 80.55 MeV/u ^12^C^6+^ beam shows that the measurement resolution to the beam center could reach 6.45 μm for unsaturated beam projections.

## 1. Introduction

Cancer seriously threatens human life and health. Heavy-ion cancer treatment is a unique form of radiotherapy. The heavy-ion beam is hailed as the ideal radiation therapy for the 21st century and is also known as the “heavy ion knife”. Because most of the energy loss of heavy ions occurs at the end of the range, a sharp energy peak—a Bragg peak—is formed [1]. When heavy ions reach the tumor site, they will deposit most of the initial energy at the tumor site, which can protect the normal tissue around the tumor to a greater extent, thereby improving the treatment effect and reducing toxic side effects. Due to its physical and biological characteristics, a heavy-ion beam is a superior tool for tumor radiotherapy. High local doses can be delivered to deep tumors by modulating the ion kinetic energy. To meet the increasing needs of cancer patients, many countries and laboratories have begun research related to heavy-ion cancer treatment and actively promoted related technologies. The pioneering heavy-ion beam cancer-treatment research originated from Lawrence Berkeley Laboratory (LBL) in the United States. LBL used its high-energy synchrotron heavy-ion accelerator to conduct clinical trials of heavy-ion beam radiation therapy in 1975. The heavy-ion cancer-treatment device HITAG of the German Heavy Ion Research Center (GSI) and the heavy-ion medical accelerator complex (HIMAC) of the National Institute of Radiological Sciences (NIRS) in Chiba, Japan, have successively allowed for clinical treatments [2,3,4]. Since 1993, the Institute of Modern Physics, Chinese Academy of Sciences, has used the medium-energy heavy-ion beam provided by the Heavy Ion Research Facility Lanzhou (HIRFL) to research the biological effects of heavy-ion radiation [5,6]. In November 2006, the first clinical treatment trial was conducted at the HIRFL superficial cancer treatment terminal. In 2019, China’s Gansu Province Wuwei Cancer Hospital (Wuwei Heavy Ion Center) installed China’s first heavy-ion therapy system with independent intellectual property rights. Since 2019 [7], it has completed heavy-ion therapy for more than 1020 patients.

To fully utilize the advantages of heavy-ion cancer treatment, the beam-monitoring system needs to monitor the heavy-ion beam in real-time to ensure that the total dose and position are consistent with the prescribed plan. The beam-monitoring system must also have low obstruction to reduce the risk of the influence of the beam. The following detector systems are typically used for therapeutic beam monitoring.

The Faraday cup is commonly used to measure the profile and intensity of beams [8,9]. This type of detector works by placing a charge collector in a cylinder with an entrance hole through which the particle beam enters. Under a negative voltage of several hundred volts, secondary electrons are guided back into the charge collector to measure the beam. The Faraday cylinder scans the proton therapy device Gantry 1 at the PSI point to complete the measurement of the beam dose with a measurement error of no more than 2% [10]. However, the Faraday cup is a blocking beam-monitoring detector, which truncates the beam at the detector and affects the beam structure.

Beam transformer detectors utilize a transformer to capture the magnetic field associated with a DC beam [11], generate the related voltage, and then process the signal using electronics. The detector is used in the Serpukhov proton synchrotron to monitor the intensity of the beam inside the accelerator and the efficiency of the ejected beam with an accuracy of 10 mA [12]. With the technology’s development, the DC-beam transformer’s energy resolution can reach the μA level. The energy resolution of the nA level has been achieved by utilizing the “low-temperature current comparator” of the superconducting sensor (SQUID) [9]. The detector can only measure beam intensity and longitudinal profile, not beam position, and is susceptible to interference from high-power pulsed devices in practical applications.

The ionization chamber detectors [13] are mainly charged particles interacting with the working gas (mainly ionization or excitation) to generate electron-ion pairs, which drift in the electric field in the sensitive volume of the detector to generate induced currents, which are processed in a certain way to obtain relevant information about the incident particles. Strip-electrode ionization chambers, pixel ionization chambers, etc., have evolved on the principle of ionization chamber detectors. 

The split strip-electrode ionization chambers [14,15] typically use strip electrodes to acquire and output a current signal that can be used to measure the beam position and two-dimensional dose distribution of carbon ions. The German Center for Heavy Ion Research (GSI) [16] uses a strip-electrode ionization chamber to monitor the position of the beam current, which is composed of a 1.5 mm-thick G10 plate with a 39 μm copper skin, and the detector has a total of two anodes, with an anode thickness of 3.079 mm. However, the disadvantage of this system is that the strip electrodes are thicker, which has a more significant impact on the beam energy dispersion and purity and results in the quality of the beam current deviating from the theoretical value. The Institute of Modern Physics of the Chinese Academy of Sciences has developed a pixel ionization chamber system successfully applied to the treatment terminal of a medical heavy-ion accelerator for real-time monitoring of beam position, profile uniformity, and other key beam quality parameters [17]. The system mainly comprises two anodes and three cathodes, with an anode made of FR4 PCB and gold-plated microstrip line and a cathode made of a 13 μm double-sided aluminum-plated mylar film. The positional resolution of the system is close to 73 μm after testing.

The pixel ionization chambers [18,19] are made with anodes in the form of small blocks similar to pixel dots, and the signals are acquired and processed to yield information on the relative beam dose distribution. The Italian Institute of Nuclear Physics (INFN) [20] has installed pixel ionization chambers in the Proton Radiotherapy Unit, with pixel blocks 5 mm^2^ in size. The front electronics of this type of detector are highly integrated, and there is no dead time in the beam current measurement since the detector has excellent sealing, effectively reducing the attenuation of the signal transmission. The system has been tested with a positional resolution close to 1.9 mm.

The TPC (time projection chamber) also utilizes the principle of charged particles passing through the interaction with the working gas to monitor the beam [21,22,23,24,25,26]. When the beam passes through the TPC, it will ionize with the inert gas inside the detector and generate electrons. Under the action of the electric field, they will drift to the anode of the detector, and the TPC can record the trajectory of the electrons. Based on the advantages of TPC detectors, we propose designing a silicon pixel chip-based TPC heavy-ion beam-monitoring system, HiBeam-T. An array of silicon pixel sensors is used as the anode of the HiBeam-T to collect electrons and measure the position of the particle beam. HiBeam-T has the characteristics of no damage to the beam, good position resolution, short response time, and fast readout speed. In this paper, the design, implementation, and characterization of HiBeam-T will be discussed.

## 2. The Detector

**Figure 1:**
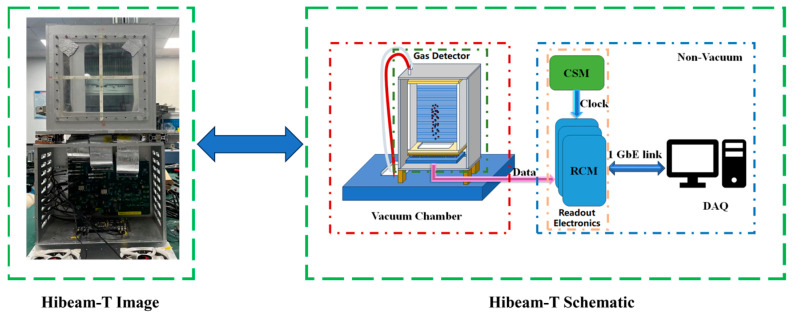
Structure of the HiBeam-T system.

Figure 1 shows the overall structure of Hibeam-T. HiBeam-T is composed of a gas detector and readout electronics. We employ the TPC detection principle in our design. The gas chamber is 30 cm × 20 cm × 40 cm, filled with gas as the working medium. Electrons are generated when the therapeutics ions pass through and ionize the gas in the chamber. The silicon pixel sensors, working as detector anodes, collect the electrons and convert them to electrical signals sent to the readout electronics outside the chamber. In total, 3 Readout Control Modules (RCMs) handle 40 silicon pixel sensors as detector channels. The first and second RCMs connect 16 sensors, and the third RCM connects 8 sensors. The readout electronics digitizes, processes, and packages the detector signals to the data acquisition system (DAQ) via three Gigabit Ethernet links. In the DAQ, the position of the incident beam is calculated.

**Figure 2:**
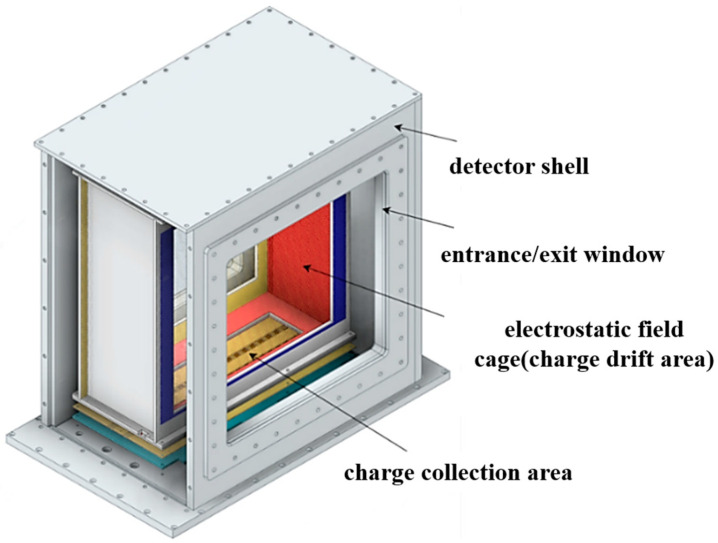
The sketch of the HiBeam-T.

Figure 2 shows the sketch of the gas chamber. It mainly consists of a detector shell with membranous entrance and exit windows, an electrostatic field cage forming the charge drift area, and a charge collection area consisting of forty silicon pixel sensors. The detector shell is constructed with aluminum alloy plates, which are glued together or sealed with an O-ring. The entrance and exit windows are 13 μm thick Kapton membranes double-side-coated with aluminum. The safe-high-voltage (SHV) and gas-flowing feed-throughs are installed on the bottom of the detector shell. The electrostatic field cage is formed by an array of Ø100 μm gold-plated tungsten (GPT) filaments connected to the metal strips on the supporting printed circuit board (PCB) frame, forming a series of concentric equipotential electrode rings connected by resistors.

**Figure 3:**
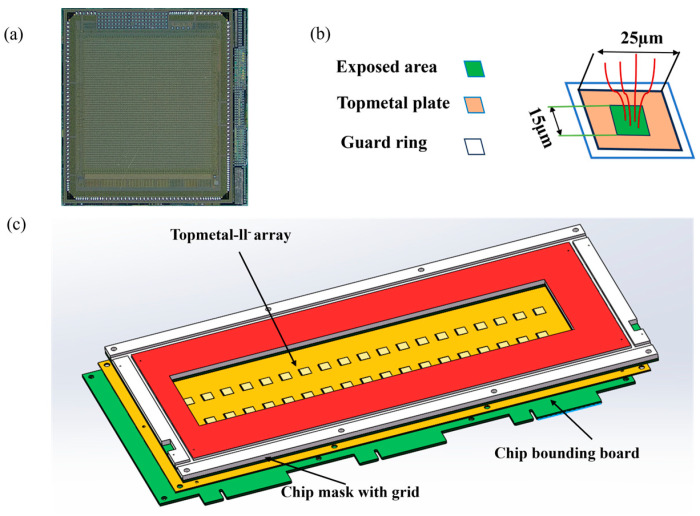
(**a**) The Topmetal-II- CMOS pixel sensors. (**b**) The Topmetal structure. (**c**) Structure of the charge collecting area.

As shown in Figure 3c, the charge collection area mainly comprises the Topmetal-II- CMOS pixel sensors array, a sensor mask, and a sensor boundary plate. The sensor mask is essentially a hollowed-out sheet electrode. The resulting apertures coincide accurately with the sensitive area of the sensor array. A GPT array is also arranged on the mask to cover the apertures. The readout plate consists of two rows of the Topmetal-II- CMOS pixel sensors [27,28,29].

Figure 3a shows the Topmetal-II- CMOS pixel sensor, which is a pixel sensor that uses standard 0.35 μm CMOS technology. Each Topmetal-II- CMOS pixel sensor has 72 × 72 pixels with a size of 83 μm × 83 μm [30]. This leads to a charge-sensitive region of 6 mm × 6 mm. Each pixel has a sensor named Topmetal to collect charge from surrounding media directly with its exposed area. As shown in Figure 3b, the total size of the Topmetal sensor is 25 μm × 25 μm, and the size of the exposed area is 15 μm × 15 μm [31].

**Figure 4:**
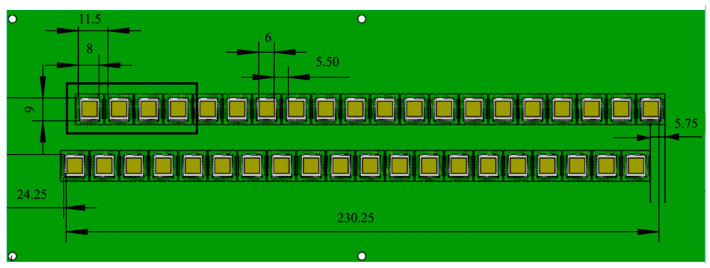
Silicon pixel sensor arrangement.

The pixel sensors are inter-distributed in the column direction, forming a 23 cm one-dimensional no-dead-zone length. To avoid the influence of inter-sensor dead zones during beam monitoring and to ensure the device’s measurement accuracy, we designed a specially arranged sensor array, as shown in Figure 4, which is two parallel rows of sensors. Taking the first row of sensor arrays as the benchmark, the second row of sensor arrays is translated 5.75 mm to the left. According to system design specifications, the sensor-to-sensor distance across the two rows of sensor arrays on the binding board is 5.5 mm.

## 3. The Readout Electronics

**Figure 5:**
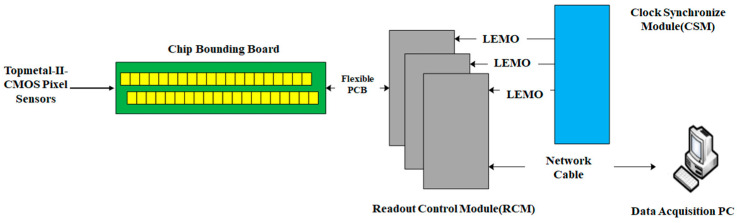
The Readout electronics.

Figure 5 illustrates the structure of the readout electronics, involving three Readout Control Modules (RCMs) and a Clock Synchronization Module (CSM). The RCM receives data and controls the Topmetal II- pixel sensors. The CSM distributes synchronized clock and control signals to the entire readout electronics. RCMs connect to the chip bounding board via flexible PCB. The data transmission between the RCM and the computer is via a Gigabit Ethernet link.

The Topmetal-II- CMOS pixel sensors convert the ionized electrons generated as the beam traverses the detector’s working gas into an electrical signal. The electrical signals travel to the Readout Control Module (RCM) for processing. Once received, the ADC on the RCM conducts data compression of the 14-bit data per input based on a predefined pattern. The compressed data transform into a 16-bit format by appending “0” to the upper two bits. After processing, the data are sent to the computer using a Gigabit Ethernet link. The computer obtains the data through the data acquisition card and then completes the data synchronization of different sensors according to the timestamps in the data. Subsequently, the frames from each sensor are assembled based on timestamps to construct a unified beam profile. Ultimately, the position of incidence of the beam is obtained based on the calculation of the center of the beam profile. The CSM ensures synchronization across the three RCMs. The hardware of the RCM and the CSM have also been used in the prototype of the beam monitor for CEE [32].

### 3.1. The Readout Control Module (RCM)

**Figure 6:**
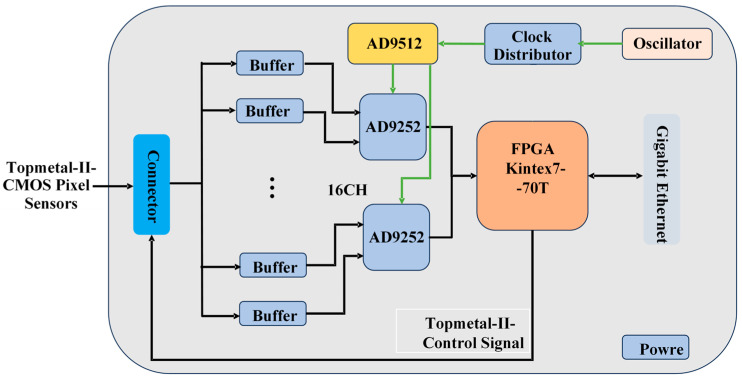
RCM block diagram.

**Figure 7:**
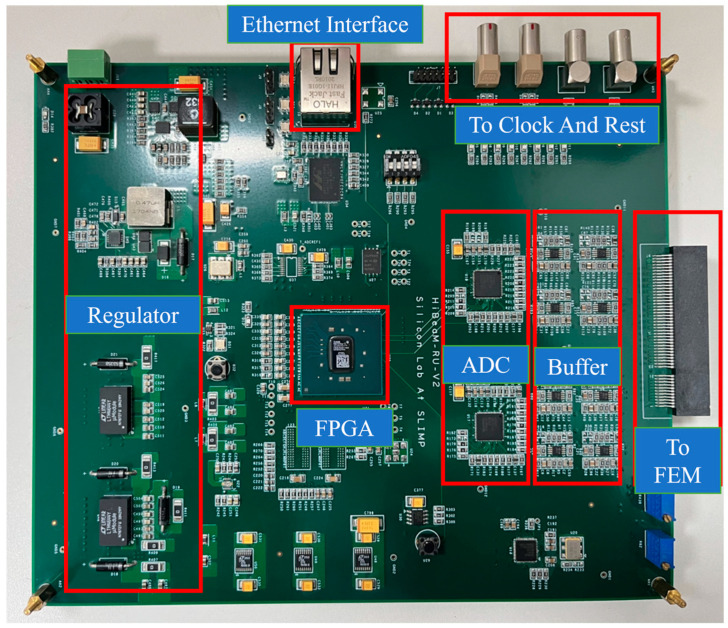
The architecture of the prototype readout electronics RCM [32].

Figure 6 and Figure 7 show a block diagram and a picture of the RCM. The RCM mainly consists of the main field-programmable gate array (Xilinx Kintex-7 FPGA), the analog buffer circuit, two high-resolution ADCs (AD9252), the power supply circuit, and the Ethernet Interface. Each RCM can receive and process signals from up to sixteen Topmetal-II- CMOS pixel sensors. The analog signal from the Topmetal-II- CMOS pixel sensors is transmitted through the connector to the analog buffer circuit, which converts the single-ended signal to a differential signal and then filters and transmits the differential signal to the ADC for digitization. The ADCs digitize the output of the analog buffer circuit with a sampling frequency of 25 MHz. The FPGA packs the data, adds a timestamp and RCM number, and communicates with the data acquisition computer via Gigabit Ethernet. At the same time, there is a current-monitoring circuit on the board, which can monitor the current of the Topmetal-II- CMOS pixel sensors in real-time.

**Figure 8:**
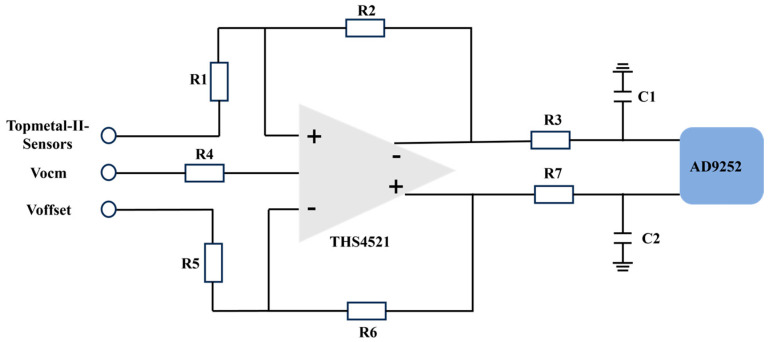
Analog buffer block diagram.

The analog buffer circuit depicted in Figure 8 consists primarily of an operational amplifier (THS4521), resistors, and capacitors, which provide a drive and buffer circuit to the ADC. A resistor is employed to supply an offset voltage (Voffset) for Topmetal-II-CMOS pixel sensors and calibrate their output baseline. The operational amplifier ensures that the AD9252 receives a common-mode voltage that aligns with its requirements.

**Figure 9:**
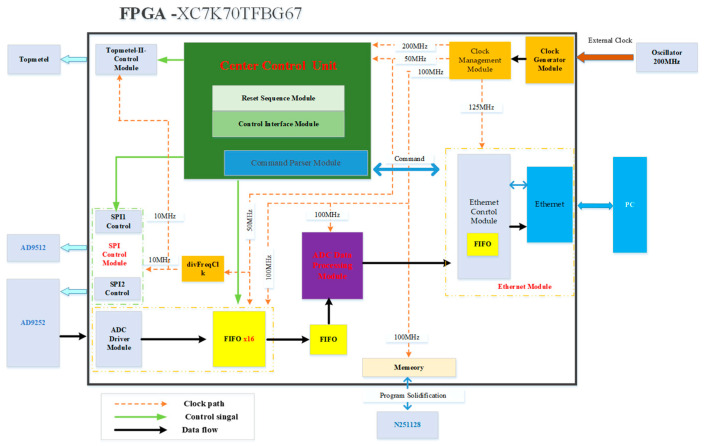
The FPGA designs.

To satisfy the control and data processing needs of the HiBeam-T system concerning the Topmetal-II- CMOS pixel sensors, the FPGA firmware incorporates crucial modules, as shown in Figure 9, These modules include the Central Control Unit, the Topmetal-II- Control Module, the SPI Control Modules, the Ethernet Module, the ADC Data Module, and the Clock Generator Module.

The Central Control Unit comprises primarily a Reset Sequence Module, a Control Interface Module, and a Command Parser Module. Specifically, the Reset Sequence Module oversees the reset operation for individual modules, ensuring their sequential transition into operational states. Initially, we convert the reset from a hardware asynchronous reset to a synchronous reset controlled by the system clock. After the AD9512 provides a stable reference clock for the other modules, we complete the configuration of the ADC and Ethernet. Finally, the control of the Topmetal-II- CMOS pixel sensors is achieved. ADC converts analog signals to digital using analog-to-digital conversion. Subsequently, the FPGA processes this digital signal, and ultimately, the system transmits the processed data to the host computer through Ethernet. The Command Parsing Module mainly interacts with the PC for information, parses the commands issued by the PC, and assigns them to the SPI Control, Topmetal-II- Control, and ADC driver. The Command Parsing Module is a control module with bi-directional transmission capabilities. The downstream function is to parse the data from the PC which can be parsed by address bits according to data-defined rules and match the appropriate registers to store the data bits. The Topmetal-II- Control Module generates control signals for the Topmetal-II- CMOS pixel sensors. Within the ADC Data Processing Module, the ADC driver converts the ADC’s 14-bit width parallel data into 1-bit width data synchronized with the ADC clock. Subsequently, the ADC Data Processing Module packages each frame of the ADC data, starting with the marker signal of the Topmetal-II- CMOS pixel sensors. The Clock Generator Module allocates a 200 MHz clock to the Central Control Unit, another 100 MHz clock to the ADC Data Module, and a 125 MHz clock to the Ethernet Module. An asynchronous FIFO resolves the clock domain crossing between the ADC Data Processing Module and Command Parser Modules. The Clock Management Module uses the IP core to convert the system clock into different frequencies to provide the working clock for each module; the off-sensor crystal provides the system clock. The Clock Generator Module provides the functional clocks for the Central Control Unit, the ADC Driver Module, the ADC Data Processing Module, and the Ethernet Module.

### 3.2. The Clock Synchronization Module (CSM)

**Figure 10:**
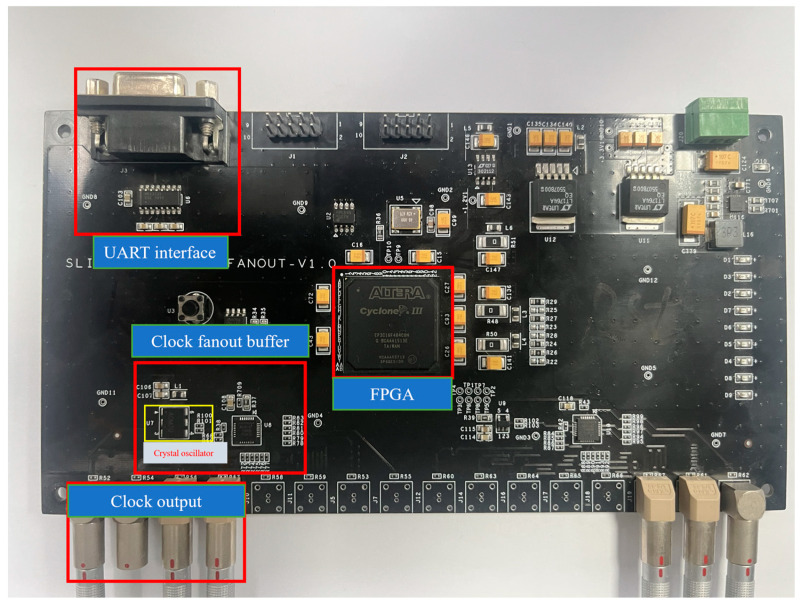
The picture of the CSM.

The CSM provides synchronous clocks and synchronization signals to the RCMs. It is essential to ensure that data collected from all RCMs are time-synchronized to reconstruct the ion track. Figure 10 shows the picture of the RCM, primarily comprising a Field Programmable Gate Array (Altera Cyclone III FPGA), a Clock fanout buffer, a 200 MHz Crystal oscillator and the UART interface. To realize the synchronization between different RCMs, the CSM uses the UART bus to realize communication with the PC. The design connects eight pairs of differential clock signals and eight pairs of differential synchronization signals to differential connectors and transmits them to the RCM via a coaxial cable. When the FPGA receives a synchronization command from the DAQ via the RS232 interface, the FPGA sends three pairs of differential synchronization signals to the RCM to restart the timestamp counting. The Clock Synchronization Module not only guarantees precise data acquisition and transmission but also provides a reliable timestamped benchmark for subsequent data analysis and processing.

## 4. Performance Test Result

**Figure 11:**
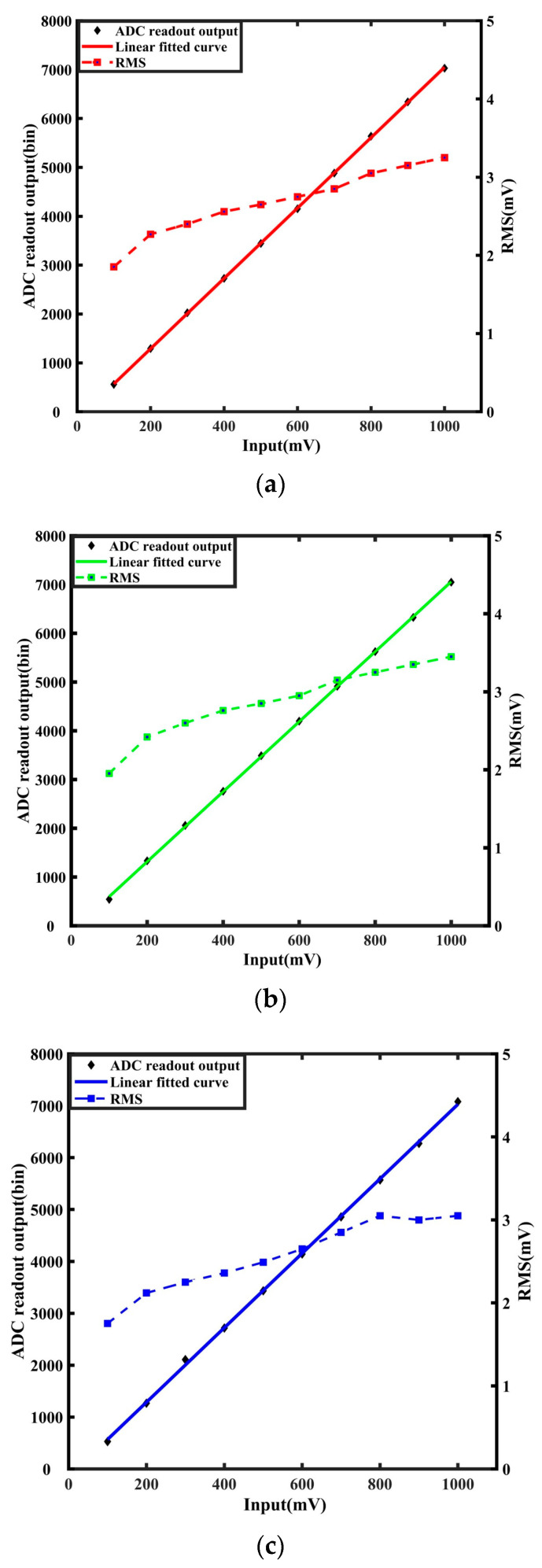

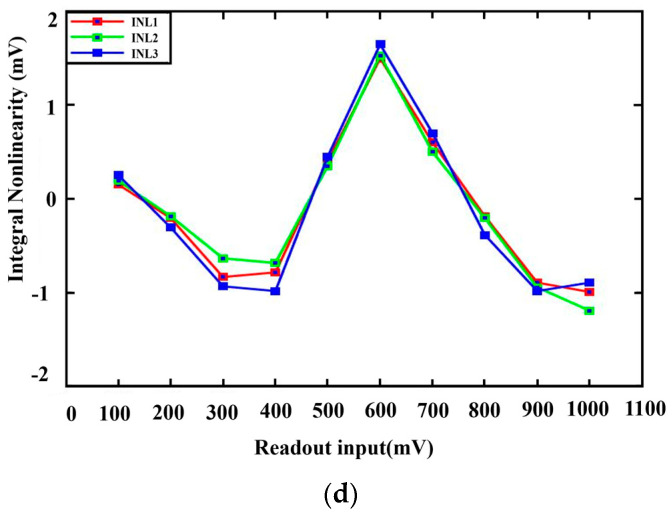
Readout electronics test. (**a**–**c**) Shows the linear function fitted with the output and the corresponding input of the RCM. (**d**) Shows the integral non-linearity (INL) with each specific input value.

The three RCMs underwent testing to assess the performance of the readout electronics system within the HiBeam-T setup. In the lab test, a precise pulse generator (Tektronix AFG3252C) provides input to the RCM, with the input signal varying from 0.1 V to 1 V in the step of 100 mV. Each RCM has 16 signal channels, and we analyzed all the channel data of each RCM. For visualization, we selected the analysis results of the data of the third channel in each RCM for presentation. The linear functions fitted to the outputs and corresponding inputs of the RCM are shown in Figure 11a–c. The average root-mean-square (RMS) noise is about 2–3 mV. Figure 11d shows each specific input value’s integral non-linearity (INL). The maximum INL is approximately 2 mv, which is 0.2% of the entire dynamic range.

**Figure 12:**
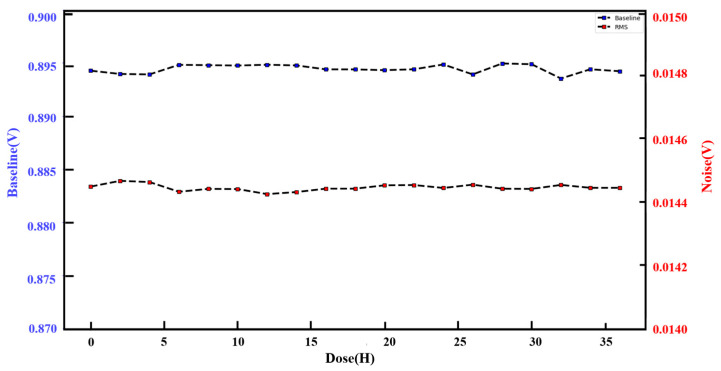
Long time systematic test.

We carried out a long-term stability test on the system to ensure it could meet the performance requirements for long-term operation at the beam terminal. We built a specialized test-bed in our lab to test the system. During this test, we did not provide any external signals to the detector. We allowed the system to run continuously for 36 h, with data collection every two hours. For an overall noise and baseline analysis of the system, we randomly selected a data packet from the collected data and intercepted 100 frames of data from the data packet; we then calculated the average value of each pixel in the 100 frames to obtain a frame of the image. Then, we performed Gaussian curve-fitting on the values of all pixels in the frame image. According to the above method, the data collected each time were analyzed, and 19 points were obtained for the baseline and noise. As shown in Figure 12, the experimental results we obtained show that the average noise level is below 0.0145 V, and the overall baseline of the system is averaging at 0.8945 V, indicating that the system’s noise level is satisfactory, allowing the system to maintain stability over long periods of operation. This prolonged system stability test ensures the system’s reliability in physical applications and provides an essential benchmark for future performance evaluation and optimization.

**Figure 13:**
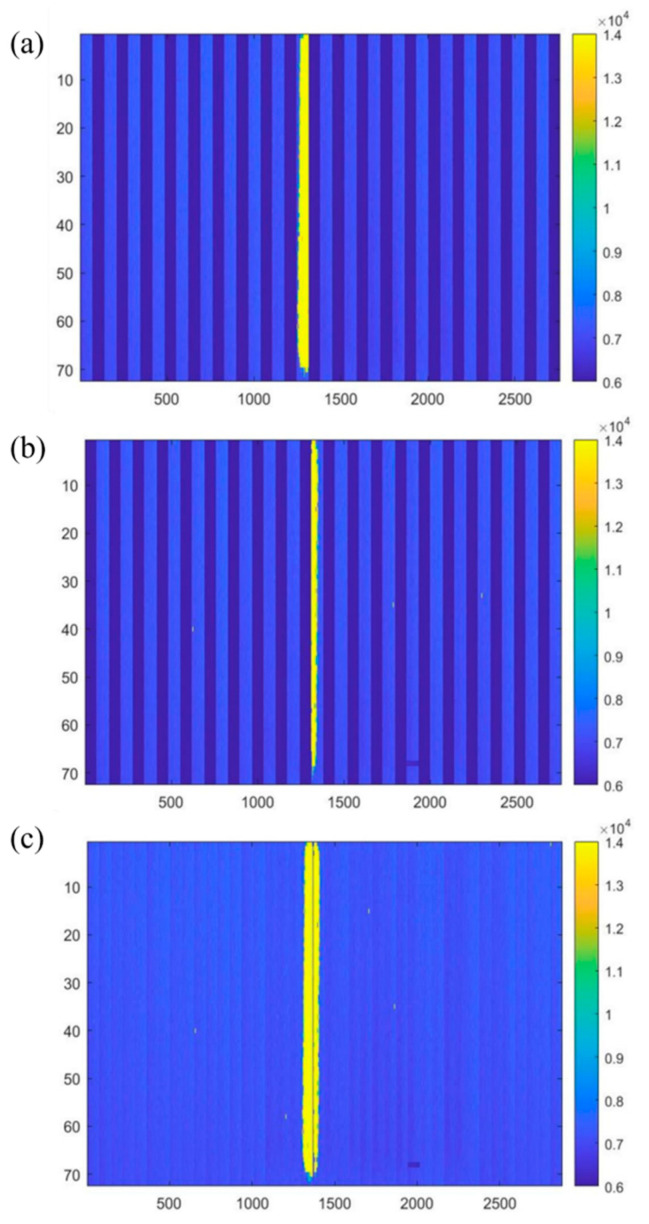
(**a**) Image reconstruction of the first row. (**b**) Image reconstruction of the second row. (**c**) Filled first row with second row.

Then, we performed an image reconstruction of a complete beam trajectory in two parts. In the first part, we performed an image reconstruction of the first row of sensor array data. Because there was 5.5 mm spacing between sensors, we filled the spacing with a 72 × 66 zeros array. The dark blue in Figure 13a represents this filled area. We subjected the second row of sensor array data to image reconstruction in the second part. We used the same image reconstruction method for the second row of sensor arrays, as shown in Figure 13b. During the complete track image reconstruction, we sorted and integrated the upper and lower rows based on the sensor arrangement order, unifying them into a single row. Each sensor in the first row overlaps with the adjacent sensor in the second row by about three columns of pixels (approximately 0.25 mm) on each edge. Hence, we removed the three columns on each edge of the sensors in the second row and then filled the trimmed image into the gap area in the first row. After this process, image reconstruction concludes, presenting the final result shown in Figure 13c.

**Figure 14:**
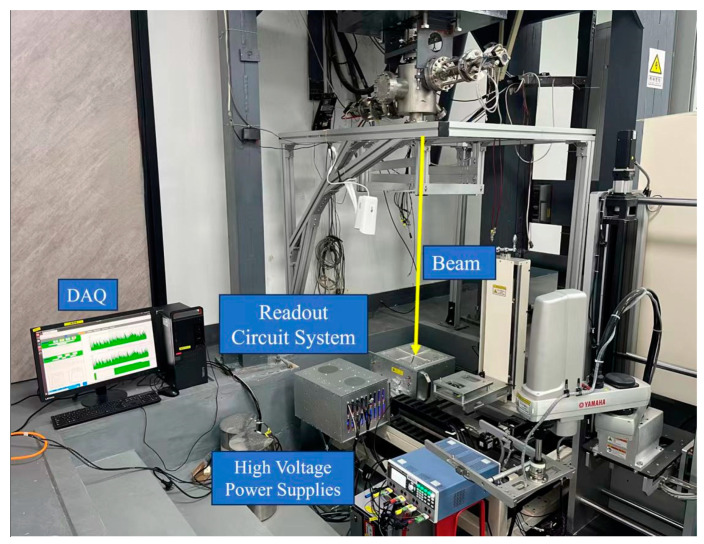
Detector testing program.

As shown in Figure 14, the beam test was carried out at the HIRFL-TR4 radiation terminal [32,33], and P10 (90% Ar and 10% CH_4_) gas was used as the working gas. A continuous 80.55 MeV/u ^12^C^6+^ Gaussian beam passed through Ø6 mm stainless steel apertures and traversed the detector perpendicularly. The beam intensity used in our experiments was 20–30 nA, which is similar to that used in Wuwei Cancer Hospital. The beam intensity of the ^12^C^6+^ beam during the experiment was about 10^6^ ion/s [34]. A voltage of −40 KV equivalent to an electric field of −150 V/cm was applied to the detector’s field cage, guiding the generated electrons toward the charge collection area.

**Figure 15:**
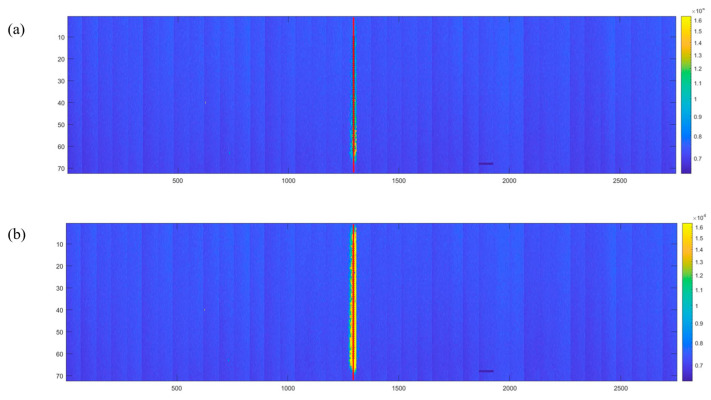
2D spectrum of ^12^C^6+^ beam. (**a**) Unsaturated 2D spectra of ^12^C^6+^ beam. (**b**) Saturated 2D spectra of ^12^C^6+^ beam.

The yellow line in Figure 15 represents an example of the measured ^12^C^6+^ beam track. The values of each column’s pixels were first subtracted by the corresponding baseline and then summed to form a one-dimensional distribution. First, along the incidence direction, the pixels in each column were summed to obtain a one-dimensional spectrum of the track, and a Gaussian fit was used to obtain the μ and Full-Width of Half Maximum (FWHM) of the one-dimensional spectrum, and the center of gravity of each row was calculated within the range of FWHM. Finally, a straight line was fitted to all the computed centers of gravity to represent the real track (red line). The value ${\Delta P}_{i}$ represents the distance from the center of mass to each row of the fitted track. Using Equation (1), the standard error (SE) of the mean value of $\bar{\Delta P}$ for all rows was calculated to be 6.45 μm, and this represents the position resolution of the beam monitor that measured the track [35,36].

(1) $$P_{reso}=\sqrt{\frac{1}{n(n-1)}\sum_{i=1}^{n}{\left(\Delta P_{i}-\bar{\Delta P}\right)}^{2}}$$

**Figure 16:**
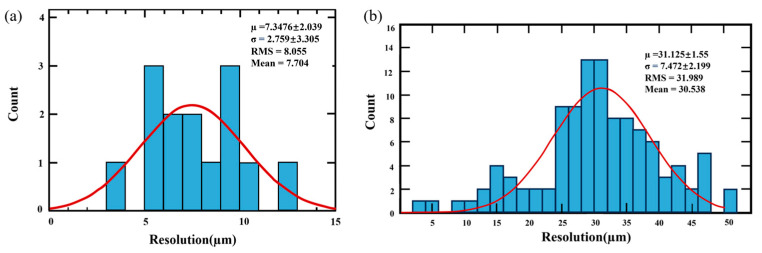
(**a**) The positional resolution of 14 ^12^C^6+^ non-saturated beam projections. (**b**) The positional resolution of 108 ^12^C^6+^ saturated beam projections.

According to the data analysis, unsaturated projects were observed at the initial phase of each beam injection, followed by eventually saturated projections. This is because with a beam flux of up to 30 nA, the charge collected by each pixel will exceed the input dynamic range of its charge-sensitive amplifier (CSA), causing the output of each pixel to be saturated. As shown in Figure 16a, the positional resolution of 14 ^12^C^6+^ non-saturated beam projections was calculated as 7.48 μm ± 2.04 μm. Subsequently, the positional resolution of 108 ^12^C^6+^ saturated beam projections was determined to be 31.13 μm ± 1.55 μm, as shown in Figure 16b.

Addressing the beam trace-saturation issue in this experiment, we will add a parallel wire gate structure above the mask in the electron collection area of the detector for the next experiment. This parallel wire gate structure will regulate the electron passage count and timing, deactivating when electrons reach the sensor’s sensitive region. Once one beam track has been processed, the gate will reopen for subsequent beams, ensuring trace-path saturation is mitigated.

## 5. Conclusions and Discussion

HiBeam-T is a time projection chamber (TPC) for monitoring the position and profile of therapeutic heavy-ion beams. The detector part is a gas chamber with forty Topmetal-II- CMOS pixel sensors as the anode to collect ionization charge. The readout electronics include three Readout Control Modules (RCMs) and a Clock Synchronization Module (CSM). The RCMs receive and handle the data from pixel sensors and then transmit them to the data acquisition system via a Gigabit Ethernet link. The CSM distributes the synchronization clock and control signals to the RCMs. Laboratory tests show that the entire readout electronics worked stably, with an overall non-linearity of less than 1%. In addition, the beam test with ^12^C^6+^ of 80 eV/u particles shows that the positional resolution for non-saturated and saturated beam projection is 7.48 μm ± 2.04 μm and 31.13 μm ± 1.55 μm, respectively. The test results indicate that HiBeam-T can be a beam monitor for the carbon ion therapy terminal.

Table 1 compares the performance of the Hibeam-T with other beam-monitoring systems, which reveals that the Hibeam-T beam-monitoring system has a great advantage in position monitoring.

## Figures and Tables

**Table 1:** Performance comparison of beam flow monitoring systems.

	Position Accuracy	Beam Parameter	Impact on Beam	Application Scenarios
Hibeam-T: TPC with Pixel Readout	6.45 μm	Vertical profile, location	Non-blocking	Potential for Wuwei heavy-ion therapy for cancer
Plit Strip-Electrode Ionization Chambers [17]	73 μm	Vertical profile, location	Non-blocking	Medical heavy-ion accelerator
Faraday Cup [10]	Energy measurement error < 2%	Intensity	Blocking	Proton therapy device
Pixel Ionization Chambers [20]	1.9 mm	Vertical profile, location	Non-blocking	Italian Institute of Nuclear Physics
Beam Transformer [12]	Energy resolution nA level	Vertical profile, Intensity	Non-blocking	Serpukhov proton synchrotron

## Data Availability

The dataset generated and analyzed during this study is available from the corresponding author upon reasonable request, but restrictions apply to the commercially confident details.

## References

[1] Schardt D., Elsässer T., Schulz-Ertner D. (2010). Heavy-ion tumor therapy: Physical and radiobiological benefits. Rev. Mod. Phys..

[2] Haberer T., Debus J., Eickhoff H., Jäkel O., Schulz-Ertner D., Weber U. (2005). The Heidelberg Ion Therapy Center. Radiother. Oncol..

[3] Li Q., Dai Z., Yan Z., Jin X., Liu X., Xiao G. (2007). Heavy-ion conformal irradiation in the shallow-seated tumor therapy terminal at HIRFL. Med. Biol. Eng. Comput..

[4] Noda K., Furukawa T., Fujisawa T., Iwata Y., Kanai T., Kanazawa M., Kitagawa A., Komori M., Minohara S., Murakami T. (2007). New Accelerator Facility for Carbon-Ion Cancer-Therapy. Radiat. Res..

[5] Liu T., Song H.S., Yu Y.H., Yan D., Sun Z.Y., Tang S.W., Lu F.H., Wang S.T., Zhang X.H., Li X.Q. (2023). Toward real-time digital pulse process algorithms for CsI (Tl) detector array at external target 292 facility in HIRFL-CSR. Nucl. Sci. Tech..

[6] Pompos A., Durante M., Choy H. (2016). Heavy Ions in Cancer Therapy. JAMA. Oncol..

[7] Xiao G., Li Q., Zhang X.Q. (2020). The development and commercialization of carbon ion cancer therapy facility. Sci. Technol. Devices TA.

[8] Brown K.L., Tautfes G.W. (1956). Faraday-cup monitors for high-energy electron beams. Rev. Sci. Instrum..

[9] Koziol H. (2001). Beam diagnostics for accelerators[R]. CERN.

[10] Lin S., Boehringer T., Coray A., Grossmann M. (2009). More than 10 years experience of beam monitoring with the Gantry 1 spot scanning proton therapy facility at PSI. Med. Phys..

[11] Unser K. (1981). A toroidal DC beam current transformer with high resolution. IEEE. Trans. Nucl. Sci..

[12] Cuperus H.J. (1973). A beam transformer system for measuring fast ejection efficiencies. Part. Accel..

[13] Alkhazov D.G., Komar A.P., Vorob A.A. (1967). Ionization fluctuations and resolution of ionization chambers and semiconductor detectors. Ncul. Instrum. Methods.

[14] Boriano A., Bourhaleb F., Cirio R., Cirrone G.A., Cuttone G., Donetti M., Garelli E., Giordanengo S., Luparia A., Marchette F. (2006). Preliminary results with a strip ionization chamber used as beam monitor for hadron therapy treatments. Nucl. Phys. B Proc. Suppl..

[15] Solovov V., Chepel V., Lopes M.I., Abrantes J., Marques R.F., Policarpo A.J. (2003). Mini-strip ionization chamber for γ-ray imaging. IEEE Trans. Nucl. Sci..

[16] Brusasco C., Cattai A., Cirio R. (1997). Strip ionization chambers as 3-D detector forhadron therapy. Nucl. Instrum. Methods A.

[17] Wei K., Xu Z., Mao R., Zhao Z., Zhao T., She Q., Kang X., Wang J., Li S., Li M. (2020). Performances of the beam monitoring system and quality assurance equipment for the HIMM of carbon-ion therapy. J. Appl. Clin. Med. Phys..

[18] La A.R., Garella A.M., Bourhaleb F. (2006). A pixel ionization chamber used asbeam monitor at the Institut Curie-Center de Protontherapie de Orsay. Nucl. Instrum. Methods A.

[19] Belletti S., Cirio R., Cocuzza L., Degiorgis P.G., Donetti M., Madon E., Marchetto F., Marletti M., Marzoli L., Peroni C. (2001). Pixel segmented ionization chamber for therapeutical beams of photons and hadrons. Nucl. Instrum. Methods Phys. Res. Sect. A.

[20] Amerio S., Boriano A., Bourhaleb F., Cirio R., Donetti M., Fidanzio A., Garelli E., Giordanengo S., Madon E., Marchetto F. (2004). Dosimetric characterization of a large area pixel-segmented ionization chamber. Med. Phys..

[21] Hilke J.H. (2010). Time projection chambers. Rep. Prog. Phys..

[22] Atwood W.B., Barczewski T., Bauerdick L.A., Bellantoni L., Blucher E., Blum W., Boudreau J., Boyle O., Cinabro D., Conway J. (1991). Performance of the ALEPH time projection chamber. Nucl. Instrum. Methods Phys. Res..

[23] Ackermann K.H., Adams N., Adler C., Aluyshin M., Ananeva M.A., Anderson M., Averichev G., Bacher A., Balewski J., Balrannikova O. (1999). The star time projection chamber. Nucl. Phys..

[24] Shane R., McIntosh A.B., Isobe T., Lynch W.G., Baba H., Barney J., Chajecki Z., Chartier M., Estee J., Famiano M. (2015). SRITa time-projection chamber for symmetry-energy studies. Nucl. Instrum. Methods A.

[25] He R., Niu X.Y., Wang Y., Liang H.W., Liu H.B., Tian Y., Zhang H.L., Zou C.J., Liu Z.Y., Zhang Y.L. (2023). Advances in nuclear detection and readout techniques. Nucl. Sci. Tech..

[26] Ren W., Zhou W., You B., Fang N., Wang Y., Yang H., Zhang H., Wang Y., Liu J., Li X. (2020). Topmetal-M: A novel pixel sensor for compact tracking applications. Nucl. Instrum. Methods A.

[27] Zhang H., Zhang Y., Yang H., Qian C., Li X., Sun X., Wang D., Huang R., Wang Y., Zhou W. (2021). Hi’Beam-A: A Pixelated Beam Monitor for the Accelerator of a Heavy-Ion Therapy Facility. IEEE Trans. Nucl. Sci..

[28] Zhang Y., Yang H., Zhang H., Sun X., Yu D., Jin Y., Feng Y., Li X., Liu J., Peng P. (2020). Design of a novel pixelated residual gas ionization profile monitor for the 320 kV high-voltage platform at IMPCAS. Nucl. Instrum. Methods A.

[29] An M., Chen C., Gao C., Han M., Ji R., Li X., Mei Y., Sun Q., Sun X., Wang K. (2016). A low-noise CMOS pixel direct charge sensor, Topmetal-II. Ucl. Instrum. Methods Phys. Res..

[30] Zou S., Fan Y., An M., Chen C., Huang G., Liu J., Pei H., Sun X., Yang P., Wang D. (2016). Test of Topmetal-II- in liquid nitrogen for cryogenic temperature TPCs. Nucl. Instrum. Methods A.

[31] Gao C., Huang G., Sun X. (2016). Topmetal-II-: A direct charge sensor for high energy physics and imaging applications. J. Instrum..

[32] Yang H., Liao J., Wang H., Gao C., Zhang H., Sun W., Li X., Zhao C. (2022). Readout Electronics of the Prototype Beam Monitor in the HIRFL-CSR External-Target Experiment. Electronics.

[33] Cai X., Xia J., Zhan W., Xu H. (2009). The commissioning progress of the cooler storage ring HIRFL-CSR in Lanzhou. JPCS.

[34] Gao Z., Zhang X., Ju Y., Chen L., Ge H., Zhang Y., Ma F., Wan T., Zhang H., Shi G. (2022). Nuclear reaction measurements of 80.5 MeV/u ^12^C beam bombarding on C, Cu, W, Au, Pb targets. Nucl. Instrum. Methods A.

[35] Yang H., Zhang H., Gao C., Wang H., Sun X., Liu J., Wang S., Li X., Ren W., Zhou W. (2021). Hi’Beam-S: A Monolithic Silicon Pixel Sensor-Based Prototype Particle Tracking System for HIAF. IEEE Trans. Nucl. Sci..

[36] Fan Y., Gao C., Huang G., Li X., Mei Y., Pei H., Sun Q., Sun X., Wang D., Wang Z. (2014). Development of a highly pixelated direct charge sensor, Topmetal-I, for ionizing radiation imaging. arXiv.

